# The impact of HER2-low status on response to neoadjuvant chemotherapy in clinically HER2-negative breast cancer

**DOI:** 10.1007/s12094-022-03062-9

**Published:** 2022-12-31

**Authors:** Wei Wang, Tingting Zhu, Hao Chen, Yongzhong Yao

**Affiliations:** Department of Breast Surgery, Nanjing Drum Tower Hospital, The Affiliated Hospital of Nanjing University Medical School, Nanjing, 210008 China

**Keywords:** Breast cancer, HER2-low expression, Neoadjuvant chemotherapy, MLH1

## Abstract

**Purpose:**

Low expression of HER2 (HER2-low expression) in breast cancer (BC) has unique biological characteristics. However, whether HER2-low expression has an impact on neoadjuvant chemotherapy (NACT) in HER2-negative breast cancer remains unclear.

**Methods:**

This study reviewed the clinicopathological data of patients with BC treated with NACT at a single hospital from January 2018 to July 2022. Baseline patient characteristics, efficacy of NACT, and survival data were compared between the HER2-0 and HER2-low groups. The impact of NACT on HER2 status also was investigated. Subgroup analyses based on hormone receptor (HR) status were performed to explore the impact of HR signaling on HER2 status during chemotherapy.

**Results:**

The progesterone receptor-positive rate in the HER2-low group was significantly higher than that in HER2-0 group. The local treatment response of the HER2-low group was worse, but the disease-free survival rate of the HER2-low group was significantly better than that of the HER2-0 group. The proportion of patients with increased HER2 immunohistochemistry score after NACT was significantly higher in the HER2-0 group. Subgroup analysis showed that the efficacy of chemotherapy in HR + patients was significantly worse than in HR− patients, and HR + patients had a higher proportion of increased HER2 immunohistochemistry score after chemotherapy. Mechanistic studies suggested that MLH1 expression loss during chemotherapy might link HR signaling and regulation of HER2 expression.

**Conclusions:**

We found that HER2-low expressing BC exhibits differential sensitivity to chemotherapy compared to HER2-0 expressing BC. The regulation of HER2 expression by HR signaling may mediate aspects of chemoresistance.

**Supplementary Information:**

The online version contains supplementary material available at 10.1007/s12094-022-03062-9.

## Introduction

Human epidermal growth factor receptor 2 (HER2)-positive breast cancer (BC) is a highly malignant tumor subtype that accounts for 15–20% of all BC. Following the success of HER2-targeting therapies, such as trastuzumab, HER2 has become an important prognostic factor [1]. In clinical practice, there is a binary distinction between HER2+ and HER2- BC [2]. A breast tumor is considered HER2 + only if it has an immunohistochemistry score (IHC) 3+ or IHC 2+ /in situ hybridization positive (ISH)+, and a diagnosis of HER2 + indicates that a patient could benefit from one year of trastuzumab therapy [3]. Tumors that are IHC 0, IHC 1+, or IHC 2+/ISH− account for 80–90% of all cases, and are considered to be HER2- BCs and generally receive similar chemotherapy regimens [4].

It is now appreciated that there is significant heterogeneity among HER2− BC, and about 45–55% of tumors that were classified as HER2− exhibit a low to moderate expression of the HER2 protein but without ERBB2 amplification [5]. The growing consensus is that such tumors should be named HER2-low expressing BC (HER2-low BC) in order to distinguish them from BC that truly lack HER2 protein expression (HER2-0 BC). Recent clinical trials have shown that antibody–drug conjugates (ADC), in particular trastuzumab deruxtecan and trastuzumab duocarmazine, may have clinical activities in HER2-low tumors [6]. This suggests that HER2-low BC might constitute a different disease category with particular clinical and biological characteristics [7]. In a pooled analysis of four clinical trials, HER2-low BC had a significantly lower pathological complete response (pCR) rate to neoadjuvant therapy than HER2-0 BC. Subgroup analysis showed that the pCR rate was significantly lower in HER2-low tumors versus HER2-0 tumors in the hormone receptor-positive (HR+) subgroup [8]. This suggests that HER2-low expression might be an important mechanism of chemoresistance, especially in HR + BC. However, specific clinical trials for HER2-low BC are limited, and it remains to be determined whether HER2-low expression has an impact on the efficacy of chemotherapy or disease prognosis.

The expression state of the HER2 gene or protein is not permanently stable, and exhibits spatial and temporal regulation when tumor cells are stimulated by external stresses [9]. Taucher et al*.* reported that the HER2 IHC score was increased in 12% of BC patients with residual tumors after neoadjuvant chemotherapy (NACT) compared to the corresponding pretreatment biopsy specimens [10]. If patients did not achieve pCR, the results of HER2 IHC on residual tumor cells are of great significance for guiding subsequent adjuvant therapy. However, the mechanisms underlying the increase of HER2 IHC score after NACT, and whether such a change may have an impact on subsequent therapeutic responses, are unclear.

Previous studies have found that a mismatch repair deficient phenotype could be developed during chemotherapy in some cancer types, and that loss of mismatch repair activity in turn could lead to chemoresistance [11]. Although mismatch repair defects are clinically important, their significance in BC is unclear [12, 13]. One recent study has demonstrated that the mismatch repair complex (including MLH1 and PMS2) loss could activate HER2 expression in ER + HER2− cells following endocrine treatment, which may cause endocrine treatment resistance [14]. It was also suggested that increased HER2 IHC score after chemotherapy in HER2− BC, especially in ER + /HER2− disease, might be regulated by mismatch repair activity loss; this hypothesis needs further validation.

In the present study, we compared the efficacy of NACT between HER2-0 and HER2-low expressing BC, and evaluated the impact of NACT on HER2 expression in residual disease. Our purpose was to demonstrate the significance of HER2-low expression in chemoresistance and to reveal the underlying mechanism that causes heterogeneous expression of HER2 during NACT.

## Methods

### Study design and population

This retrospective study included subjects clinically diagnosed with HER2− BC who underwent NACT followed by surgery at the Nanjing Drum Tower Hospital between January 2018 and July 2022. This study was approved by the ethical and scientific committee of the Nanjing Drum Tower Hospital, and informed consent was obtained from all patients.

Inclusion criteria included: (1) age > 18 years old; (2) invasive BC confirmed by biopsy; (3) at least immunohistochemical results for ER, PR, HER2, and Ki67 were available; (4) HER2 IHC score 0, 1+, or 2+/ISH−; (4) all patients should meet the NACT criteria according to established guidelines [15]; and (5) radical surgery for each patient performed at Nanjing Drum Tower Hospital after NACT.

Exclusion criteria included: (1) patients with clinical stage IV or history of previous malignant tumor; (2) clinicopathological data were insufficient or clinically diagnosed HER2+ (IHC 3+ or IHC 2+ /ISH+); (3) patients who did not meet the NACT criteria or terminated chemotherapy prematurely; (4) patients who did not undergo radical surgery at Nanjing Drum Tower Hospital after NACT.

Medical electronic records and pathology reports of all patients were reviewed. The recorded information included patient age, BMI, menstrual status, clinical stage before chemotherapy (tumor size, axillary lymph node status, distant metastasis or not), pathological results of biopsy and radical surgical specimens, NACT regimens received, imaging assessment results during NACT, Miller–Payne grading system results, and surgery methods used.

### Pathology

Pathological assessment for HR, HER2, and Ki67 was performed on biopsy and radical surgical specimens in the central laboratory of Nanjing Drum Tower Hospital. HER2 status was determined according to ASCO/CAP guidelines [16]. A HER2-low tumor was defined as having IHC 1+ or IHC 2+ /ISH−. HER2-0 was defined by IHC 0. HR+ was defined if the percentage of estrogen receptor and/or progesterone receptor-positive cells in the sample were ≥ 1%.

### MLH1 expression analysis

Paraffin sections were deparaffinized at 65 ℃ for 1 h and treated in a pressure boiler with EDTA for 10 min. The sections were placed in 3% hydrogen peroxide solution and incubated at room temperature for 10 min to block endogenous peroxidase. Each section was washed three times with phosphate-buffered saline (PBS), sealed with Protein Block Serum-free for 20 min, and incubated with primary rabbit monoclonal anti-MLH1 (Abcam, Cambridge, USA) overnight at 4 °C. After that, the sections were incubated with secondary antibody (Dako, California, USA) for 30 min, and washed with PBS three times. Tissues were stained for 5 min with fresh DAB solution, counterstained with Mayer's hematoxylin, and mounted using Permount (Fisher Scientific, New Jersey, USA). Samples were evaluated by one independent observer using an optical microscope. Only cancer cells with a distinct brown staining of the nucleus were considered MLH1 positive.

### Survival analysis

Recurrence and survival data were obtained by telephone follow-up. Disease-free survival (DFS) was defined as the time from last treatment (surgery) until any distant or local recurrence assessed by Response Evaluation Criteria in Solid Tumors 1.1 (RECIST 1.1) [17]. Overall survival (OS) was defined as the time from diagnosis to death, irrespective of cause of mortality.

### Statistical analysis

Statistical analyses were performed using SPSS 20 (IBM Corp., Armonk, NY). Continuous variables were expressed as the mean ± standard deviation, and categorical variables were expressed as percentage (%). Chi-square test or Fisher's exact test were used to compare categorical parameters between two groups. T-tests were used to compare normally distributed continuous variables. Correlation analysis of categorical parameters was performed by the Pearson *χ*^2^ test. The Kaplan–Meier method was used to plot survival curves and the log-rank test was used to compare differences in survival between groups. A *p*-value of < 0.05 was considered statistically significant.

## Results

### Patient characteristics

A total of 148 patients receiving NACT were included in this study. Fifty-five patients belonged to the HER2-0 group and the rest belonged to the HER2-low group. The clinicopathological information of patients are shown in Table 1. There were no significant differences in baseline characteristics between the two groups, except to PR-positive rate (HER2-0 group: 41.82% vs HER2-low group: 66.67%, *p* < 0.01). All patients were eligible for NACT according to the guidelines and 94.59% of patients received TAC/TEC regimens. In the HER2-0 group, albumin-bound paclitaxel was used in 24.45% of patients, liposomal doxorubicin was used in 20.00% of patients, and a combination of the two drugs was used in 34.55% of patients. In the HER2-low group, the corresponding proportions were 22.58, 17.20, and 25.80%, respectively. However, while specific drug treatments varied, there was no significant difference in drug selection between the HER2-0 and HER2-low groups. After NACT, all patients received radical surgery, including breast-conserving surgery or mastectomy. If axillary lymph node biopsy confirmed metastasis before chemotherapy, axillary lymph node dissection was performed directly, otherwise, sentinel lymph node biopsy was performed. There was no statistical difference in the surgical method used between the two groups. For patients who accepted breast-conserving surgery or had positive axillary lymph nodes, postoperative radiotherapy was required.

**Table 1:** Patient clinicopathological information

	HER2-0	HER2-low	*p*
Median age (range)	47 (27–69)	50 (26–71)	0.08
Menopausal status			
Pre/perimenopausal	36	51	0.23
Postmenopausal	19	42	
BMI	24.02 ± 3.76	24.08 ± 3.57	0.93
*T* (cm)			0.97
≤ 2	9	14	
2–5	37	63	
≥ 5	9	16	
Axillary lymph node			0.21
Negative	11	9	
Positive	34	65	
Uncertain	10	19	
Histologic grade			
I–II	32	63	0.29
III	23	30	
HR			
ER ±	31/24	64/29	0.16
PR ±	23/32	62/31	< 0.01
Ki67			
<30%	6	16	0.35
≥30%	49	77	
NACT regimen			0.36
Albumin-bound paclitaxel + liposomal doxorubicin + cyclophosphamide	19	24	
Docetaxel + anthracycline + cyclophosphamide	10	25	
Albumin-bound paclitaxel + anthracycline + cyclophosphamide	14	21	
Docetaxel + liposomal doxorubicin + cyclophosphamide	11	16	
Others	1	7	
Surgery method			0.42
Modified radical mastectomy	40	78	
BREAST-conserving surgery + SLNB	3	3	
Mastectomy + SLNB	7	8	
Breast-conserving surgery + ALND	5	4	

*SLNB* sentinel lymph node biopsy, *ALND* axillary lymph node dissection, *BMI* body mass index

### Comparisons of chemotherapy efficacy

All patients completed the standard courses of NACT. During chemotherapy, imaging assessments were performed every two cycles and all patients obtained good therapeutic effects without severe side effects. After chemotherapy, 16.36% of patients in the HER2-0 group and 12.90% of patients in the HER2-low group achieved pCR; this was not a significant difference. Histopathological responses were assessed using the Miller–Payne grading system (MP). The proportion of MP 5 was 20.75% in the HER2-0 group, 15.22% in the HER2-low group, but the difference was not significant. Since the number of residual tumor cells was small for patients with MP 4, these patients were also considered to have good responses to chemotherapy. And then, the proportion of MP 4–5 in the HER2-0 group was significantly higher than in the HER2-low group (41.82 vs 25.81%, *p* = 0.04).

Patients also were divided into HR+ and HR− subgroups according to IHC results. Subgroup analysis showed that the pCR rate of HR- patients was higher than that of HR+ patients, whether in the HER2-0 group (*p* = 0.03) or the HER2-low group (*p* = 0.04). Similar results were also obtained when local treatment responses were compared between the HR+ and HR− groups (Table 2).

**Table 2:** NACT efficacy compared between HER2-0 and HER2-low groups

	HER2-0	HER2-low	*p*
Non-pCR	46	81	0.63
pCR	9	12	
MP			0.04
1–3	32	69	
4–5	23	24	

	HR+	HR−	*p*	HR+	HR−	*p*
Non-pCR	29	17	0.03	59	22	0.04
pCR	2	7		5	7	
MP			< 0.01			< 0.01
1–3	24	8		53	16	
4–5	7	16		11	13	

*MP* Miller–Payne grading system, *pCR* pathological complete response

### HER2 expression evolution from pre- to post-NACT

Expression of HER2 protein or gene can change in response to chemotherapy. Therefore, this study also investigated the HER2 status in residual tumor cells after NACT and compared the post-NACT result to the IHC score of the same patient’s sample prior to chemotherapy. We found that 29.73% of patients had an increase of HER2 IHC score after chemotherapy. Subgroup analysis showed that the HER2 score increased in 40.00% of patients in the HER2-0 group and 23.66% in the HER2-low group (*p* = 0.04). Moreover, we also found that HR + patients had a higher proportion of HER2 IHC score increase than HR− patients, whether in the HER2-0 group (*p* = 0.05) or in the HER2-low group (*p* = 0.04) (Table 3).

**Table 3:** Comparison of HER2 IHC score before and after NACT

	HER2-0	HER2-low	*p*
HER2 score increase	22	22	0.04
Other	33	71	

	HR+	HR−	*p*	HR+	HR−	*p*
HER2 score increase	16	6	0.05	19	3	0.04
Other	15	18		45	26	

### MLH1 expression analysis

**Fig. 1:**
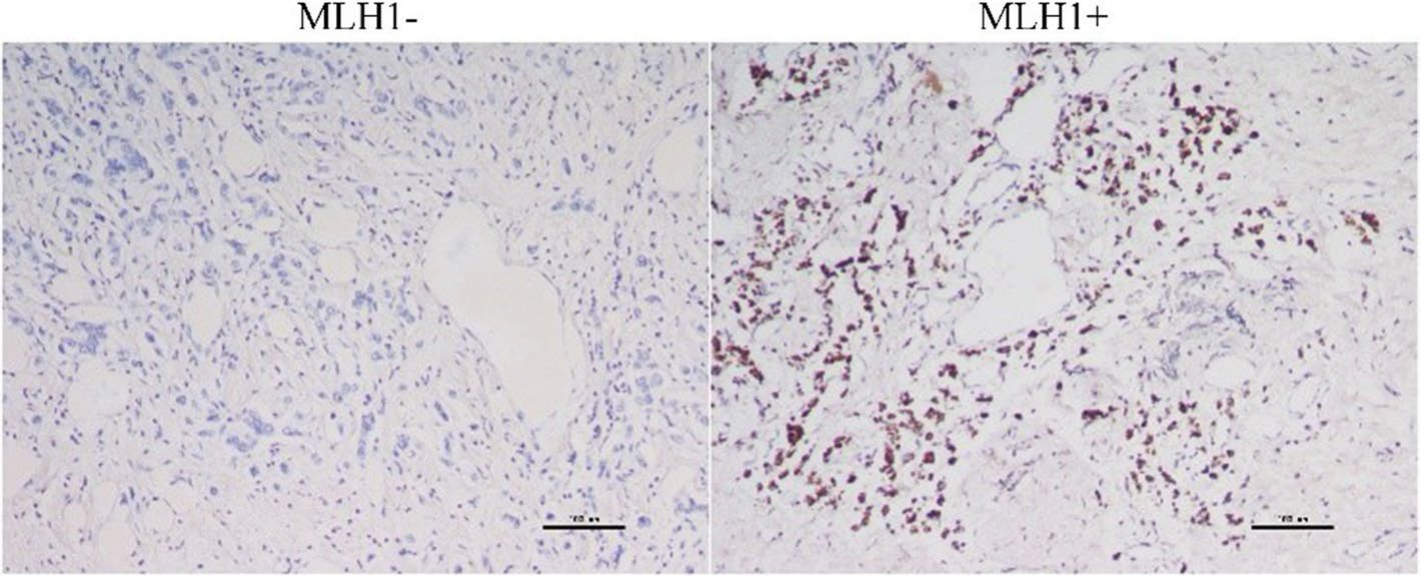
Representative immunohistochemical staining results for MLH1 in residual lesions

MLH1 expression was preliminarily analyzed on excised tissue sections (including cancer and para-cancer tissues) of 44 patients who did not achieve pCR (Fig. 1). MLH1 expression was found in 18.18% of patients. Among these, only 5% of patients with increased HER2 scores were MLH1 positive, while 29.17% of patients without increased HER2 scores were MLH1 positive (*p* = 0.05). Besides, only 9.68% of samples in the HR + group were MLH1 positive, which was significantly lower than that in the HR− group (38.46%, *p* = 0.04) (Table 4).

**Table 4:** Comparison of MLH1-positive rates in residual lesions after NACT

	MLH1+	MLH1−	*p*
HER2 score increase	1	19	0.05
Other	7	17	
HR+	3	28	0.04
HR−	5	8	

### Survival analysis

**Fig. 2:**
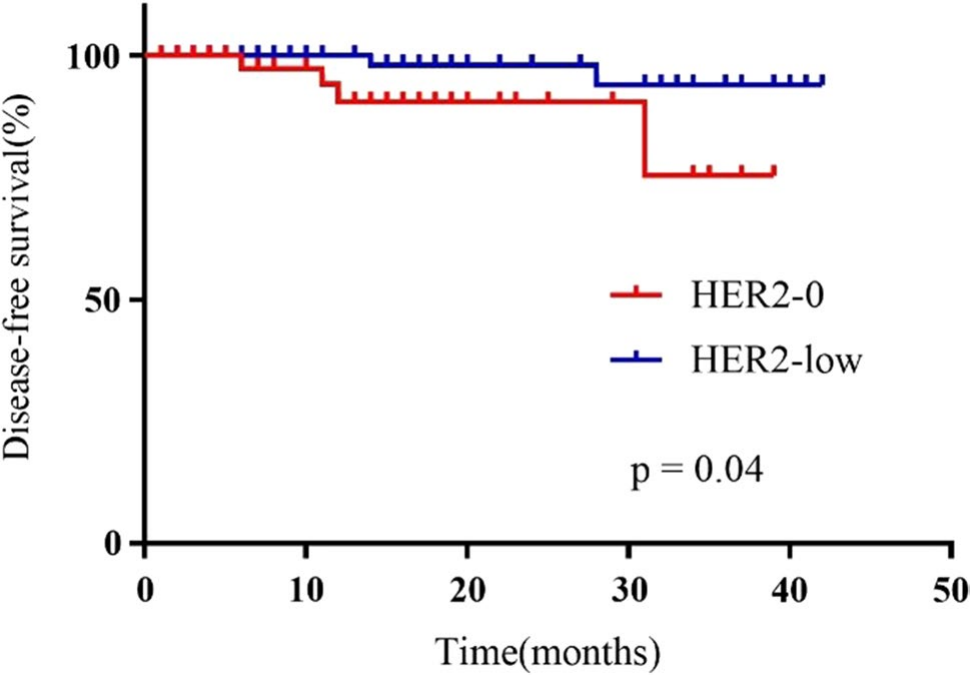
Kaplan–Meier curves of disease-free survival based on HER2-low and HER2-0 status

The median follow-up time was 18.0 months (range 0.5–42.0 months). During the follow-up period, there were no deaths observed. A total of 6 cases (4.05%) had local recurrence or distant metastasis, including 4 cases (7.27%) in the HER2-0 group and 2 cases (2.15%) in the HER2-low group. Survival analysis showed that patients in the HER2-low group had longer DFS than patients in the HER2-0 group (HR 0.16, 95% CI 0.03–0.93, *p* = 0.04) (Fig. 2).

## Discussion

New generations of ADC drugs are expected to be effective in treating HER2-low BC, which has garnered increasing attention in recent years [18, 19]. This retrospective study analyzed the clinicopathological data and therapeutic responses of BC patients with HER2-low and HER2-0 expression who received NACT. We found that pathologically confirmed HER2-0 and HER2-low BC cases accounted for ~ 50% of all patients. Among them, HER2-0 patients accounted for about 37%, and more than 60% of patients had HER2-low tumors. This was similar to previously reported proportions [20]. Previous study showed that HER2-low BC was similar to HER2 + BC, which was characterized by large tumor size, high tissue grade, high proliferation rate, and axillary lymph node metastasis [21]. In the present study, we found no significant differences in baseline characteristics between patients with HER2-0 and HER2-low BC, except for the PR positivity rate. We speculate that this was mainly related to the fact that all patients received NACT in this study, and therefore had similar clinical and pathological characteristics.

HER2 overexpression is an important factor in promoting resistance to chemotherapy [22]. In this study, the pCR rates of both the HER2-0 and the HER2-low groups were significantly lower than the pCR rate of HER2 + BC reported in the literature [23], indicating that the lack of an effective therapeutic target for HER2-0 or HER2-low BC remains a barrier to the effectiveness of NACT. Although there was no significant difference in pCR rates between the HER2-0 and HER2-low groups, the proportion of patients with MP 4–5 in the HER2-0 group was significantly higher than that in the HER2-low group. This demonstrates that tumors lacking HER2 expression had better local treatment responses than HER2-low expressing tumors [15], and it suggests that low expression of HER2 may contribute to NACT resistance. A retrospective study reported that the pCR rate in HR+ /HER2-low BC was significantly lower than in HR+ /HER2-0 BC [8]. Indeed, cross-activation between the HER2 and HR signaling pathways has been reported [24]. In the present study, we found no differences in the efficacy of NACT between HR-/HER2-0 and HR-/HER2-low groups, nor between the HR+/HER2-0 and HR+/HER2-low groups. We speculate that the lack of significant differences in NACT responses between these groups was due to the small sample size. However, we found that therapeutic effect of NACT in patients with HR- BC was better than in patients with HR + BC, regardless of whether the patients were in the HER2-0 group or HER2-low group. This is consistent with the established fact that NACT responses of triple negative BC (TNBC) are superior to those of luminal B (HER2-) subtype BC [25].

Gene expression analysis suggests that HR signaling is the main driver of biological behaviors of HER2-low BC [26]. Whether HR signaling contributes to drug resistance by regulating HER2 expression remains unclear. The spatio-temporal regulation of HER2 expression is heterogeneous, and HER2 expression becomes even more inconsistent after chemotherapy [27, 28]. It was reported that 47% of HER2-0 BC patients had an increased IHC score at recurrence, particularly in the HR+ group [29]. These tumor cells scattered across the tumor bed tend to be more resistant to therapy. Although discordance in HER2 expression has been studied extensively, no clear promoting factor has come to surface [30]. In this study, we investigated the change of HER2 expression in residual tumor cells after NACT, and found that a higher proportion of patients in the HER2-0 group had an increased IHC score after chemotherapy. Besides, we found that HR + tumors were more likely to show increased HER2 expression after NACT, whether in the HER2-0 group or in the HER2-low group. This indicates that HER2-0 tumor cells are more susceptible to the influence of HR signaling during NACT. Our data also suggest that it is important to retest the HER2 status of residual tumor cells after NACT, as HER2-low BC might be the ideal candidate of anti-HER2 ADCs in the future. Furthermore, it is important to explore the regulatory mechanism between the HR and HER2 signaling pathways [31].

Mismatch repair deficiency can be developed during chemotherapy, and the loss of mismatch repair activity promotes chemotherapy resistance in tumor cells [11, 32]. MLH1 is the most abundantly expressed mismatch repair protein, and loss of MLH1 protein expression is associated with adverse outcomes of BC. Among premenopausal patients, loss of MLH1 expression has been reported to account for 41.6% of BC cases, which implies that hormones play an important role in regulating expression of mismatch repair proteins [33, 34]. Recently, Punturi et al*.* demonstrated that the loss of MLH1 protein expression activates HER2 expression in ER + HER2− BC cells [14]. Therefore, we speculated that the loss of MLH1 protein expression during NACT might be an intermediate link in the regulation of HER2 by HR signaling. IHC analysis of MLH1 expression in residual tumor cells after chemotherapy confirmed this hypothesis. We found that the positive expression rate of MLH1 in HR + tumors was significantly lower than that in HR− tumors after chemotherapy. Furthermore, tumors with MLH1 expression loss had a higher proportion of increased HER2 IHC score. This finding supports the idea that crosstalk between the HER2 and HR pathways might play a crucial role in biologically defining the HER2-low phenotype, and the MLH1 expression status might be a crucial intermediate link. The regulatory mechanism underlying the interactions between MLH1, HR signaling, and HER2 expression merits further evaluation.

The prognostic value of HER2-low status in BC remains debatable. A retrospective study showed HER2-low status did not affect survival outcomes of patients with metastatic BC undergoing first-line treatment with endocrine therapy plus palbociclib [35]. Another study reported that patients with HER2-low BC survived significantly longer than patients with HER2-0 BC in the overall population (*p* = 0.004) and HR+ subgroup (*p* = 0.011) [36]. In this study, there was no mortality during follow-up, but the DFS was better in the HER2-low group. This seems to contradict our previous conclusion that patients in the HER2-0 group responded better to NACT. This result was verified by a recent independent study, which also found that the HER2-0 group had a higher proportion of patients who achieved pCR (14.7% versus 9.8%, *p* = 0.003), and the HER2-low patients had better 5-year OS and DFS rates (*p* < 0.001 for OS; *p* = 0.002 for DFS) [37]. In our study, we speculate that this was related to the higher proportion of TNBC in the HER2-0 group (HER2-0 group: 43.64% vs HER2-low group: 31.18%). TNBC is characterized by being more aggressive and sensitive to cytotoxic drugs. Although pCR is closely associated with a favorable long-term prognosis, TNBC is still more likely to relapse and metastasize during early follow-up, due to the lack of specific therapeutic targets to control tumor growth, especially among patients who do not achieve pCR [38]. In addition, a higher proportion of patients in the HER2-0 group had increased HER2 IHC score after NACT, suggesting that the tumors of these patients were more likely to express HER2 gene or protein after chemotherapy, which might accelerate tumor recurrence and metastasis.

This study has several limitations. First, this is a single-center retrospective study with a small sample size and short follow-up time. Even so, this study produced some meaningful results. For example, this study found that mismatch repair deficiency may be an important factor leading to variations in HER2 expression and resistance during NACT. This may suggest where to begin to explore mechanisms of drug resistance in HER2-low BC. Second, HER2 status in this study was defined based on IHC and ISH results in pathological reports. HER2 IHC staining and score interpretation could be affected by the formalin-fixation variables, artificial factors, or intra-tumoral heterogeneity [29], which might influence the conclusions of the study. Besides, even in HER2 IHC 0 BC, a small number of tumor cells (<10%) may stain weakly positive for IHC according to current testing criteria. An attempt to define HER2-low tumors using quantitative reverse transcriptase-polymerase chain reaction was challenging, as it found that HER2 mRNA levels were comparable between HER2 IHC 0 and HER2 IHC 1+ tumors, and were significantly lower than IHC 2+/ISH− tumors [39]. This suggests that there is heterogeneity in HER2 expression in HER2-low BC. How best to accurately define HER-0 and HER2-low tumors needs further optimization methods.

## Conclusion

This study found that the local treatment responses to NACT of primary lesions among patients with HER2-low BC were significantly worse than among patients with HER2-0 BC. During NACT, HR signaling may regulate HER2 expression by inducing the loss of MLH1 expression, which can lead to chemoresistance.

## Supplementary Information

Below is the link to the electronic supplementary material.

Supplementary file1 (XLSX 34 KB)

## Data Availability

The data could be obtained from the corresponding author upon reasonable request.
